# A Narrative Review of Cardiovascular Health in Autistic Individuals: Epidemiologic Evidence, Mechanistic Pathways, and Future Directions

**DOI:** 10.1007/s40471-026-00389-7

**Published:** 2026-03-30

**Authors:** Emily Hotez, Lanxin Song, Yixin Yang

**Affiliations:** 1 Department of General Internal Medicine-Health Services Research, David Geffen School of Medicine, University of California, Los Angeles (UCLA), Los Angeles, CA USA; 2 Department of Epidemiology, Fielding School of Public Health, University of California, Los Angeles (UCLA), PO Box 951772, Los Angeles, CA 90095-1772 USA

**Keywords:** Autism, Cardiovascular disease, Cardiometabolic health, Life course, Health disparities, Stress

## Abstract

**Purpose of Review:**

Autistic individuals—representing one in 31 individuals in the U.S.—experience disproportionate poor cardiovascular health. This review synthesizes the current evidence on cardiovascular health in autism, with attention to epidemiology, modifying factors, mechanistic pathways, and implications for future research and practice.

**Recent Findings:**

Evidence from population-based cohorts and systematic reviews demonstrates elevated cardiovascular risk factors among autistic individuals, driven by interacting behavioral, biological, psychosocial, and environmental pathways and modified by individual and family characteristics. Studies are limited by methodological challenges and future research should: (1) focus on comprehensive assessments of cardiovascular health, (2) align with developmental science, (3) integrate multiple levels of analysis, (4) evaluate associations between mental and physical health, and (5) shift from autism biomarkers to health biomarkers.

**Summary:**

Cardiovascular health is an urgent priority for autistic individuals and there is a need for improved research to inform effective health interventions for this population.

## Introduction

One in 31 individuals in the U.S. is autistic [1]. This population experiences disproportionate poor cardiovascular health, relative to non-autistic individuals. They also experience premature mortality, with an average 16-year lower life expectancy [2–4].

According to the American Heart Association “Life’s Essential 8”, *cardiovascular health* is multilevel and encompasses *health behaviors* and *health factors* [5]. Health behaviors include adhering to an eating pattern based on whole foods, engaging in physical activity, avoiding nicotine, and getting adequate sleep. Health factors include managing weight, controlling cholesterol, and managing blood sugar. Improved cardiovascular health lowers the risk for *cardiovascular disease (CVD)*, including coronary heart disease, stroke, and heart failure [6].

**Fig. 1:**
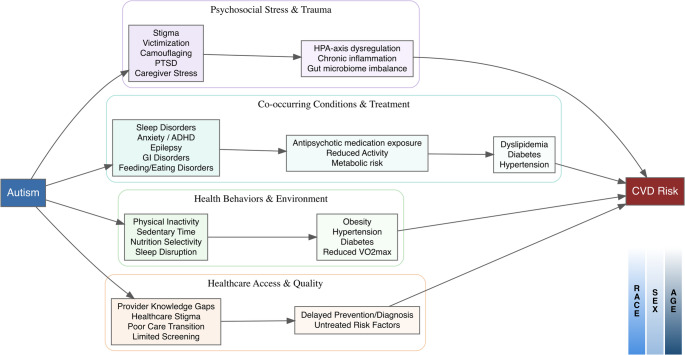
Conceptual model of pathways linking autism to cardiovascular disease risk and outcomes

The current paper summarizes the state of the evidence on cardiovascular health and CVD among autistic individuals across the life course, with a focus on epidemiology, modifying factors, mechanistic pathways, and future directions for research and practice. Key studies are summarized in **Table **1**.** The conceptual model for the current paper—derived from the literature—is presented in **Fig. **1.

## Epidemiological Evidence for Cardiovascular Risk in Autistic Populations

Evidence for poor cardiovascular health in autistic individuals has increased during the last decade and is supported by systematic reviews and prospective cohort studies. A recent systematic review of baseline data of intervention studies reporting the prevalence of cardiovascular risk factors among autistic children and adults found that—across 34 studies and 276,173 autistic participants—autism was associated with greater odds of developing overall diabetes as well as type 1 and type 2 diabetes specifically. Autism was also associated with an increased risk of dyslipidemia and heart disease [7].

Another systematic review of eight studies—with a total of 70,503 autistic participants—identified that this population experiences a greater likelihood of metabolic syndrome and type 2 diabetes [8]. The largest cohort study to date—based on a Dutch registry of 8,690,286 individuals aged 12–65 years—found that autistic people had higher risks of hypertension, dyslipidemia, diabetes, stroke, and heart failure beginning adolescence [9].

Additional studies support these findings. Analysis of a U.S. sample of Medicare beneficiaries aged 65 + found elevated prevalence of heart disease in autistic individuals (54% compared to 37%) [10]. Research has identified a causal relationship between autism and increased risk of stroke, partially mediated by type II diabetes [11]. Taken together, there is robust evidence that this population requires support for cardiovascular health.

## Autism and Cardiovascular Health: Mechanistic Pathways

### **Co-occurring Conditions**

For all populations, cardiovascular health is multifactorial. Specifically, factors contributing to cardiovascular outcomes do not function in isolation but accumulate and interact as complex systems [12]. With respect to autism specifically, a recent systematic review and meta-analysis reviewed existing guidelines, diagnostic manuals, experts, carers, and autistic people. This review encompassed 340 publications with approximately 590,000 participants. Findings revealed numerous co-occurring conditions that could potentially contribute to poor cardiovascular health among autistic participants, including developmental coordination disorder, sleep-wake problems, gastrointestinal problems, ADHD, anxiety disorder, overweight/obesity, feeding and eating disorders, elimination disorders, disruptive behaviors, and somatic symptoms and related disorders [13]. Another systematic review of 24 individual systematic reviews similarly identified multiple co-occurring conditions that are highly prevalent among autistic individuals, including sleep problems, epilepsy, sensory impairments, atopy, autoimmune disorders, and obesity [14]. The evidence suggests multiple co-occurring conditions that could potentially contribute to CVD.

### **Physical Activity**

Autistic people experience barriers to physical activity, a critical preventive strategy for CVD [15]. A U.S.-based study of autistic children and adolescents identified that—relative to non-autistic participants—autistic participants engaged in less frequent strenuous or moderate physical activity and reported less positive attitudes toward physical activity; less perceived control or ease of performing physical activity; and more barriers to physical activity [16]. Other barriers to physical activity for autistic children include bullying, challenges in community programs, and the prioritization of therapeutic interventions for this population [17]. Physical activity is a lifelong challenge for autistic individuals, with autistic adults noting the need for additional support in engaging in physical activity as well as sensory issues that prevent them from participating [18].

Barriers to physical activity may contribute directly or indirectly to cardiovascular health in this population. As an example, a cross-sectional study of autistic adults revealed that higher sedentary time was significantly associated with increased odds of high blood pressure, stroke, and precursors to mental health conditions [19].

Physical activity interventions are limited by small sample sizes. A systematic review of four physical activity interventions—spanning 149 autistic participants ranging in age from 6 to 29 years—identified that physical activity interventions had mixed effects on cardiovascular health. Specifically, interventions yielded moderate effects on VO₂max and baseline heart rate, although they showed mixed effects on participant BMI, waist circumference, and lipid profiles [20].

### **Sleep**

Autistic individuals experience sleep difficulties—including low sleep efficiency, prolonged sleep latency, increased number and length of night awakenings, and overall increased nocturnal activity [21]—which has been found to contribute to CVD in the general population [22]. In a cross-sectional study of 545 autistic adults recruited through the Simons Foundation, poor sleep quality was associated with an increased likelihood of having an overweight/obesity classification [23]. There are few well-designed sleep intervention studies targeting cardiovascular health in autistic individuals [24].

### **Nutrition**

Nutrition is an important predictor of cardiovascular health and healthy aging more generally [25]. Autistic individuals experience unique nutrition challenges, particularly related to sensory perceptions—including hyper-reactivity or hypo-reactivity to sensory input, differential visual or textural perceptions of food, and/or distinct patterns of odor detection and identification—that can affect nutritional status [26].

Findings from a randomized controlled trial with 67 autistic children and adults revealed that a combination of nutritional supplements and additional nutritional treatments shows promise in improving the nutritional profiles of this population [27]. A systematic review of 316 studies found, however, that while dietary interventions and nutritional supplements offer specific benefits, there is a need for tailored interventions that reflect collaborations between healthcare providers, educators, and families to improve population health for autistic individuals [28]. To be sure, nutritional interventions that align with developmental science are increasing; a recent virtual nutrition program for autistic adolescents and young adults demonstrated decreased mean added sugar intake as well as participant self-efficacy [29]. Yet, most nutritional interventions are small and short-term.

A notable gap in nutrition interventions in autism is that the majority target autism traits or behaviors, rather than cardiovascular health [30]. A systematic review identified 19 randomized controlled trials of nutrition interventions; interventions included gluten/casein-free diets, digestive enzymes, or camel’s milk to mitigate “challenging behaviors.”

### **Stress and Trauma**

In non-autistic populations, chronic stressors—cumulative stressful experiences over the life course—lead to cardiovascular risk factors such as obesity because they elicit psychological and physiological chronic stress responses that promote increased food consumption and fat storage [31–39].

Few studies test psychosocial or stress mechanisms to poor cardiovascular health among autistic individuals, even though they experience disproportionate chronic stressors in the form of lifelong stigma, victimization, rejection, and a reliance on camouflaging autistic traits to appear more “typical” [40–48]. To date, evidence linking stress to cardiovascular health in autism is mixed. A recent cross-sectional study of autistic children and their parents examined the association of stress—using hair cortisol concentrations (HCC)—with child and parent mental health. Autistic children had higher HCC relative to their peers, although their HCC was not linked to mental health, eating behavior, or BMI [49].

Notably, *caregiver* stress is also a potential pathway by which autistic individuals experience poor cardiovascular health [50]. As an example, caregiver stress has been found to be associated with child emotional under- and over-eating [49]. Chronic stress of caregivers evaluated by increased HCC was positively associated with obesity measures among children with disabilities [51]. Biological studies demonstrate that caregiver distress can lead to dysregulation of the hypothalamic–pituitary–adrenal axis, a pro-inflammatory state of the immune and central nervous systems, and gut microbiome imbalance [52]. Taken together, these findings suggest that stress within autistic individuals’ developmental ecosystem may be a viable intervention target.

Autistic adolescents and young adults also experience higher rates of Post Traumatic Stress Disorder (PTSD) relative to their non-autistic counterparts [53]. There are several pathways by which PTSD can impact cardiovascular health, including creating increased risk for myocardial infarction [54, 55]. Future work is needed to explore measurement of stress and trauma and elucidate their precise associations with adverse health outcomes.

### **Healthcare Access and Utilization**

Primary prevention of CVD in healthcare is essential for cardiovascular health [56]. In general, people with disabilities experience numerous barriers to healthcare access and utilization across the life course [57], resulting from multiple pathways. In particular, there is a well-documented lack of provider training regarding supporting autistic patients [58, 59]. As an example, a recent systematic review of 13 studies representing 2,706 primary care providers revealed that providers have inadequate knowledge about autism [60]. Further, many providers lack the healthcare transition tools and strategies needed for children with special healthcare needs [61].

Importantly, addressing healthcare barriers for autistic individuals is not simply a matter of providing more training; autistic patients report experiencing stigma in healthcare settings [62], which can, in turn, lead to healthcare avoidance or lack of receipt of high-quality care. In addition, autistic individuals describe the acquisition of healthcare self-advocacy skills and knowledge as a “journey” [63]. These barriers and challenges have important implications for cardiovascular health, which requires consistent, high-quality preventive care to identify, diagnose, and treat conditions proactively.

### **Other Mechanisms**

The current list of mechanisms is by no means exhaustive. As an example, antipsychotic medication usage has been associated with increased likelihood of diabetes and obesity in autistic children and adults [23, 64–66]. Between the ages of 15 to 30, BMI increases are particularly pronounced among autistic individuals who take antipsychotic medications [65]. Beyond medication usage, researchers have posited that because autism and obesity are polygenic conditions and the genetic basis of these diseases is not well-defined, there is a need for further investigation of potential molecular pathways linking obesity and autism [67].

## Modifying Factors to Cardiovascular Health in Autistic Populations

A range of individual and family characteristics have been found to modify (or moderate) cardiovascular health in autistic populations.

### **Race and Ethnicity**

Analysis of U.S. Medicaid data from 2008 to 2012 revealed that autistic beneficiaries who were Black, Hispanic, and Asian had higher odds of diabetes, hospitalized CVD, and hypertension [68]. These disparities also exist in non-autistic populations, driven primarily by the social determinants of health. The authors of this research note that autistic racial and ethnic minority adults may be at a “double disadvantage” due to their membership in multiple minority groups.

### **Sex and Gender**

There are inconclusive findings regarding the impacts of sex and gender on cardiovascular health in this population. Overall, autistic women demonstrate heightened risk of various health conditions as well as high levels of healthcare utilization [69].

There are multiple hypotheses to this finding. The “double disadvantage hypothesis” that suggests that autism and gender are minority statuses that can interact and compound to create health disparities. There is also the possibility that biological differences lead autistic women to have more chronic or severe conditions relative to men. Alternatively, autistic women may experience more challenges with the autism diagnostic process relative to men; they may also camouflage their autistic traits at greater rates. They may, therefore, experience differential treatment in the healthcare system and require more health visits before a correct diagnosis or course of treatment is determined. This may partially explain why autistic women experience disproportionate cardiovascular conditions and prediabetes, relative to non-autistic females [70].

Other findings in the literature suggest that autistic *men* may be particularly at risk for CVD, relative to women. Specifically, they have been found to be more likely than autistic women to have high blood pressure, even though autistic men and women have been found to have similar rates of high cholesterol, diabetes, and overweight/obesity, with men showing a higher average number of CVD risk factors than women [71]. Importantly, there is a general gap in research with respect to non-binary autistic individuals. Taken together, sex and gender may moderate cardiovascular risk through both biological and social pathways, although findings remain inconclusive.

### **Developmental Factors**

In all populations, cardiovascular health and risk are linked to life course development. As an example, one of the most prevalent conditions in autistic adolescents and young adults is obesity [72], with rates increasing during this time period [73–77]. For autistic individuals, this developmental period is also accompanied by higher prevalence of hypertension and type 2 diabetes [77, 78] as well as psychiatric and other medical conditions [79]. As a result, interventions administered prior to or during adolescence and young adulthood may be optimal.

## Methodological Challenges in Research of Cardiovascular Health Among Autistic Individuals

Cardiovascular health interventions designed for autistic individuals remain sparse and of low quality. As an example, a meta-analysis of 12 weight management intervention studies found only one “high-quality” study, few included subgroup analyses and female participants, and just half reporting positive results [80]. The reviewed studies featured physical activity interventions (*n* = 4), pharmaceutical interventions (specifically focusing on metformin [*n* = 2]), and “comprehensive” interventions (*n* = 6), which included nutrition, physical activity, and motivational components (e.g., opportunities for social interaction, goal setting, and family involvement). Lack of robust interventions may be due to a range of methodological challenges in the literature.

First, most studies of CVD in this population specifically assess obesity [67, 74, 81–87]. To be sure, these studies overwhelmingly find that autistic individuals have disproportionate incidence of obesity, a critical cardiovascular health precursor. Studies find, however, that body mass index (BMI) can be an insufficient cardiovascular health indicator in this population since it is not consistently associated with key biomarkers of cardiovascular health [88].

Additional challenges in CVD research in autistic populations relate to CVD research more generally. There is a need for longitudinal studies, as cardiovascular outcomes often take a significant period of time to develop [89]. Similarly, there is significant between-study variation in reported findings across the life course; specifically, CVD researchers cite a need to report child and adult outcomes separately [89]. As with analyzing CVD in all populations, there are numerous confounding factors for CVD, particularly related to the social determinants of health [89]. Finally, self- or caregiver-reported height and weight is prone to error [67].

Finally, many biomarker studies in autism focus on identifying biomarkers for *autism*, rather than *CVD* biomarkers. Although research testing autism biomarkers may support efforts to receive earlier diagnoses and interventions [90], there remains a need to increase focus on identifying mechanisms that support the health and quality of life of autistic individuals.

## Next Steps and Recommendations for Research

1. Shift from individual risk factors to comprehensive cardiovascular health. Although studies of individual risk factors such as BMI provide important insight into cardiovascular risk, future research should prioritize measurement of overall cardiovascular health. Ideally, this work would integrate behavioral, clinical, and biomarker data and generate findings that are directly translatable to clinical practice and intervention development.
2. Align cardiovascular research with developmental science. Assessing cardiovascular health during sensitive developmental periods—such as adolescence and young adulthood—may enhance the relevance of findings for intervention among at-risk populations. Additionally, research should prioritize longitudinal designs, as examining cardiovascular health across the life course can clarify temporal ordering and potential causal pathways. Studies should also examine key moderators, including race/ethnicity, sex/gender, and co-occurring conditions, to ensure representation of diverse autistic individuals.
3. Advance multilevel studies and interventions. Currently, research emphasizes the need for interventions that address barriers to and facilitators of receipt of preventive services for people with disabilities [91]. These interventions would be multilevel—ideally integrating healthcare system reform, provider training, and individual empowerment and self-advocacy [92]. In practice, a systems approach would promote access to care coordination services, create environmental or procedural changes that can make care more accessible and efficient, and increase family support [93]. There is a particular need for interventions during developmentally sensitive periods (e.g., the transition to adulthood) when autistic adults often experience fragmented healthcare [94, 95].
4. Expand research on stress, trauma, and mental health in relation to cardiovascular health. Future studies should include comprehensive biological and psychological assessments of both acute and chronic stressors, as well as co-occurring mental health conditions. Autistic populations experience disproportionate exposure to stress and trauma, and links between stress and cardiovascular health have been established in non-autistic populations. This line of research should inform the development and testing of mental health interventions that also promote physical health.
5. Reorient biomarker research toward accessibility and quality of life outcomes. Biomedical research is typically inaccessible for autistic individuals who often experience heightened stress, anxiety, and discomfort related to research participation [96, 97]. Accessibility in research is multifaceted and includes: (1) physical accessibility (e.g., sensory-friendly environments and accommodations for communication differences); (2) intellectual accessibility (e.g., plain language materials); and (3) social accessibility (e.g., non-stigmatizing language) [98]. Future studies should address gaps in research accessibility. Additionally, much of biomedical research in autism has focused on identifying biomarkers of autism itself, rather than biomarkers that can inform strategies to improve health and quality of life. Future biomarker research should prioritize outcomes that directly support well-being and long-term health among autistic individuals.

## Conclusion

Autistic individuals experience elevated cardiovascular risk across the life course, driven by interacting individual and environmental mechanisms. The existing literature demonstrates epidemiological evidence of disproportionately poor cardiovascular health yet remains limited by methodological factors. Advancing cardiovascular health for autistic populations will require a paradigm shift toward comprehensive, developmentally informed, and multilevel research that prioritizes longitudinal designs, inclusive and accessible methods, and outcomes that meaningfully improve health and quality of life.

## Key References

- Dhanasekara CS, Ancona D, Cortes L, Hu A, Rimu AH, Robohm-Leavitt C, et al. Association Between Autism Spectrum Disorders and Cardiometabolic Diseases: A Systematic Review and Meta-analysis. JAMA Pediatr. 2023 Mar 1;177(3):248–57.
  - ○  This study featured a systematic review and meta-analysis and aimed to examine the association between autism and cardiometabolic diseases in a systematic review and meta-analysis. Across 34 studies, autism was associated with a greater risk of diabetes, dyslipidemia, and heart disease. Autistic children were at a greater associated risk of developing diabetes and hypertension compared with adults.
- Chieh AY, Bryant BM, Kim JW, Li L. Systematic review investigating the relationship between autism spectrum disorder and metabolic dysfunction. Res Autism Spectr Disord. 2021 Aug 1;86:101821.
  - ○  This systematic review aimed to examine metabolic dysfunction and type 2 diabetes mellitus among autistic individuals across eight studies. Higher prevalence for metabolic syndrome components among autistic individuals was observed. There was a high degree of methodological heterogeneity across studies.
- Li Y, Xie T, Li L, Lin J, Vos M, Chang Z, et al. Cardiometabolic conditions in people with autism: a nationwide prospective cohort study from the Netherlands. Nat Ment Health. 2026 Jan;4(1):157–64.
  - ○  This study featured a large cohort study, using Dutch register data of 8,690,286 individuals aged 12-65 years. Autism was associated with a higher rate of hypertension, dyslipidemia, stroke, and heart failure. Associations were observed in adolescent, young, and middle-aged adults, but not older adults.
- Matheson BE, Douglas JM. Overweight and obesity in children with autism spectrum disorder (ASD): A critical review investigating the etiology, development, and maintenance of this relationship. Rev J Autism Dev Disord. 2017;4(2):142–56.
  - ○  This review aimed to examine the prevalence rate of overweight and obesity among autistic children. Findings revealed that autistic children are at higher risk for excess weight gain, relative to their non-autistic counterparts.
- Micai M, Fatta LM, Gila L, Caruso A, Salvitti T, Fulceri F, et al. Prevalence of co-occurring conditions in children and adults with autism spectrum disorder: A systematic review and meta-analysis. Neurosci Biobehav Rev. 2023 Dec 1;155:105436.
  - ○  This systematic review aimed to estimate the prevalence of co-occurring conditions in autistic children and adults. The most common co-occurring conditions were developmental coordination disorder, sleep-wake problem, gastrointestinal problem, ADHD, anxiety disorder, overweight/obesity, feeding and eating disorder, elimination disorder, disruptive behavior, and somatic symptoms and related disorder.
- Rydzewska E, Dunn K, Cooper SA. Umbrella systematic review of systematic reviews and meta-analyses on comorbid physical conditions in people with autism spectrum disorder. Br J Psychiatry J Ment Sci. 2021 Jan;218(1):10–9.
  - ○  This umbrella systematic review aimed to improve understanding of co-morbid conditions among autistic individuals. Across 24 studies, findings revealed that sleep problems, epilepsy, sensory impairments, atopy, autoimmune disorders, and obesity are common.
- Charlier L, Cordeiro L, Neto JLC, Signini ÉDF, Barbosa-Silva J, Corbellini C, et al. Effects of Physical Exercise on Cardiometabolic Health in Individuals with Autism Spectrum Disorder: A Systematic Review. Healthcare [Internet]. 2025 Feb 17 [cited 2026 Jan 13];13(4). Available from: https://www.mdpi.com/2227-9032/13/4/439
  - ○  This systematic review aimed to investigate the effects of physical exercise on cardiometabolic health in autistic individuals. Across four studies, interventions demonstrated mixed effects, with improvements in BMI, waist circumference, and lipid profiles in some cases and moderate effects on VO2max and baseline heart rate.
- van der Lubbe A, Swaab H, Vermeiren R, van Rossum EFC, van Balkom IDC, Ester WA. Chronic Parenting Stress in Parents of Children with Autism: Associations with Chronic Stress in Their Child and Parental Mental and Physical Health. J Autism Dev Disord [Internet]. 2025 Feb 21 [cited 2026 Jan 14]; Available from: 10.1007/s10803-025-06736-9.
  - ○  This study aimed to understand the associations between chronic stress and mental and physical health of parents of young autistic children. Across 181 parents of 99 young autistic children, parental hair cortisol concentration was associated with child hair cortisol concentration.
- Gilmore D, Krantz M, Weaver L, Hand BN. Healthcare service use patterns among autistic adults: A systematic review with narrative synthesis. Autism. 2022 Feb 1;26(2):317–31.
  - ○  This systematic review aimed to understand how often autistic adults use five important healthcare services, relative to non-autistic adults. Across 16 articles, findings revealed that autistic adults most often had equal or higher use of services. Frequent emergency department visits and hospitalizations may reflect that these services are not adequately meeting autistic adults’ needs.
- Mohammedsaeed W, El Shikieri AB, Alharbi MF. Exploring the link between exercise, BMI, cardiometabolic health, glycemic control, and health outcomes in autistic children: A comprehensive investigation. Res Autism. 2025 Sep 1;127:202665.
  - ○  This study aimed to analyze the health outcomes of autistic children, focusing on exercise habits, BMI, cardiometabolic markers, glycemic status, and lifestyle behaviors. Results showed substantial variability in health profiles and no significant correlations between BMI and biochemical markers. Strong and moderate correlations were observed within cholesterol markers.

## Data Availability

No datasets were generated or analysed during the current study.

## Appendix

**Table 1:** Recent studies on cardiovascular disease and cardiometabolic risk in autistic populations (2000-2025) in alphabetical order (First author)

Author (year, study)	Analytical design	Sample size	Exposure	Outcome	Measure of association	Unit for effect estimation	Effect estimate (95% confidence interval or other measures)
CVD Outcomes							
Dhanasekara, et al., 2023	Systematic review & meta- analysis	34 studies; 276,173 with ASD; 7,733,306 without ASD	ASD vs non‑ASD status	Diabetes (overall, T1DM, T2DM), dyslipidemia, atherosclerotic macrovascular disease, heart disease, hypertension, stroke, and peripheral vascular disease among individuals with autism	Relative risk (RR)	Ratios are unitless but comparing ASD vs non‑ASD (binary variables)	Diabetes overall: RR 1.57 (1.23–2.01); T1DM: RR 1.64 (1.06–2.54); T2DM: RR 2.47 (1.30–4.70); dyslipidemia: RR 1.69 (1.20–2.40); heart disease: RR 1.46 (1.42–1.50); hypertension: RR 1.22 (0.98–1.52); stroke: RR 1.19 (0.63–2.24)
Li et al., 2025	Prospective cohort study	8,690,286 individuals aged 12-65 years (111,795 with ASD and 8,578,491 without ASD)	ASD vs non‑ASD status	First incidence of cardiometabolic conditions and specific outcomes (hypertension, dyslipidemia, diabetes, stroke, heart failure)	Hazard ratio (HR)	Ratios are unitless but comparing ASD vs non‑ASD (binary variables)	Cardiometabolic condition: HR 1.20 (1.18–1.23); Hypertension: 1.16 (1.14–1.19); Dyslipidemia: 1.17 (1.12–1.23); Diabetes: 1.22 (1.14–1.30); Stroke: 1.23 (1.14–1.34); Heart failure: 1.28 (1.07–1.53); The highest increased risk was observed in ASD and eating disorders after adjusting SES 2.03 (1.77-2.33)
Chieh et al., 2021	Systematic review	8 studies; 70,503 with ASD; 2,281,891 without ASD	ASD vs non‑ASD status	Metabolic syndrome and its components (hyperglycemia, hypertension, dyslipidemia), T2DM	Mainly prevalence ratios/ORs per primary studies (not pooled)	Ratios are unitless but comparing ASD vs non‑ASD (binary variables); biomarkers: mg/dL (or mmHg for BP); incidence rate: per 1,000 person-years	Not pooled; Higher prevalence/incidence/odds of hyperglycemia, hypertension, dyslipidemia, and incident/prevalent T2DM in ASD
Cardiometabolic Precursors							
Gilmore et al., 2022	Systematic review	4 studies; total N = 40,876 autistic adults	Overweight/obesity (BMI-defined)	Mortality, hospitalizations, emergency department (ED) visits, healthcare costs, chronic health conditions, quality of life, social participation/interactions, and self-esteem	Odds ratios, hazard ratios, regression coefficients (as reported in included studies)	Number of in-hospital mortality; having the health conditions or not; obese vs. not obese per BMI category/unit (study-specific)	In-hospital mortality: OR 2.24 (1.44–3.46); Type II diabetes: HR 2.87 (1.37–6.00); Other outcomes showed increased comorbidity burden or null associations (CIs not consistently reported)
Micai et al., 2023	Systematic review and meta-analysis	340 studies; ≈590,000 individuals with ASD	ASD population (no comparator)	Point prevalence of overweight/obesity and 37 other point, 27 lifetime co‑occurring conditions (CCs)	Pooled prevalence (proportion); risk of bias assessment results	% of ASD individuals with condition	Developmental coordination disorder had highest overall point prevalence among mental health/psychiatric CCs (87% [95% CI 87-88%]); Motor problem was highest among medica and neurological CCs (40% [19-64%]); Pooled estimated of prevalence for overweight/obesity: 33% (95% CI 25–41%), and for metabolic disorder: 3% (95% CI 1–5%)
Rydzewska et al., 2021	Umbrella systematic review of systematic reviews	24 studies (children and adults)	ASD vs general population	Physical comorbidities, including obesity, in autism	Various (RR, OR, prevalence ratios, etc. across included reviews)	% of ASD individuals with condition; % for pooled prevalence; other units vary for different systematic reviews	Not pooled; obesity, sleep problems, epilepsy, atopy, autoimmune disorders more prevalent in ASD
Mechanistic Pathways							
Matheson & Douglas, 2017	Systematic/critical narrative review of observational studies	31 studies; total sample sizes ranged from small clinical samples to >65,000 youth with ASD (ages 2–20 years)	ASD in children and adolescents	Prevalence rates of overweight and obesity, or both among children and adolescents	Prevalence rates, odds ratios, and descriptive comparisons (in reviewed studies), rate comparison for children with ASD and typically developing children	% for rate of overweight or obesity or both	Most studies (80%) reported equal or higher prevalence of overweight/obesity in children with ASD compared with peers; selected studies reported increased obesity risk (e.g., OR ≈1.4–5.0), though confidence intervals were not consistently reported
Mohammedsaeed et al., 2025	Cross-sectional	~150 autistic children aged 2–15 years	Physical activity (exercise frequency/type), BMI	Age at diagnosis, anthropometric measures, and biochemical profiles	Pearson correlation coefficients (r and r²)	Per unit BMI (kg/m²) and biochemical markers (mg/dL and %); PE were reported in times/week	Reported PE indicates a preference for low-impact, repetitive activities; No significant associations between BMI and LDL (r = −0.02), HDL (r = −0.06), total cholesterol (r = −0.12), triglycerides (r = −0.11), glucose (r = −0.11), or HbA1c (r = 0.07); strong correlation between LDL and total cholesterol (r = 0.91, p < 0.0001), and same for triglycerides and total cholesterol (r = 0.34, p < 0.0001); Significant correlation between HbA1C and glucose levels.
Charlier et al., 2025	Systematic review	4 studies; 149 participants aged 6-29 years with ASD	Physical exercise (PE)	Cardiometabolic disorders and modifiable risk factors, such as total cholesterol, HDL-C, LDL-C, HR, glucose levels, BMI, triglycerides, and non-traditional risk factors	Mean change / mean difference (continuous)	Units of all outcome variables (weight: kg; BMI: kg/m²; waist circumference: cm; cholesterol, glucose, and triglycerides: mg/dL or mmol/L; VO₂max: mL·kg⁻¹·min⁻¹; HR: ppm)	In most studies, BMI: up to −2.8 kg/m²; waist circumference: up to −1.86 cm; lipid profiles improved in some studies; VO₂max and resting HR showed moderate gains
Van der Lubbe et al., 2025	Cross-sectional	102 children with ASD and their parents (101 mothers and 86 fathers)	Stress measured using hair cortisol concentrations (HCC) and self-reported parenting stress (OBVL)	Child mental health, eating behavior, and physical health	χ² statistics and Spearman’s r	Per 1 pg/mg increase in HCC for Spearman’s r	25.3% vs reference group: 2.5% (χ² = 8.92, p < 0.01); Negative association between maternal HCC and both externalizing and internalizing problem behavior, and total problem behavior (r = −.22-−.29, p <.05); Positive association between reported stress of mothers and both externalizing and internalizing problem behavior, total problem behavior, and both emotional overeating and overeating (r =.22-.42 p <.05); Positive association between reported stress of fathers with reported most autism symptoms, both externalizing and internalizing problem behavior, and total problem behavior (r =.24-0.49, p <.05).

## References

[1] Shaw KA. Prevalence and early identification of autism spectrum disorder among children aged 4 and 8 years — Autism and developmental disabilities monitoring network, 16 sites, United States, 2022. MMWR Surveill Summ. 2025;74. 10.15585/mmwr.ss7402a1. 10.15585/mmwr.ss7402a1 PMC12011386 40232988

[2] Hirvikoski T, Mittendorfer-Rutz E, Boman M, Larsson H, Lichtenstein P, Bölte S. Premature mortality in autism spectrum disorder. Br J Psychiatry. 2016;208(3):232–8. 10.1192/bjp.bp.114.160192. 26541693 10.1192/bjp.bp.114.160192

[3] Croen LA, Zerbo O, Qian Y, Massolo ML, Rich S, Sidney S, et al. The health status of adults on the autism spectrum. Autism. 2015;19(7):814–23. 25911091 10.1177/1362361315577517

[4] Baraskewich J, von Ranson KM, McCrimmon A, McMorris CA. Feeding and eating problems in children and adolescents with autism: A scoping review. Autism. 2021;25(6):1505–19. 10.1177/1362361321995631. 33653157 10.1177/1362361321995631 PMC8323334

[5] www.heart. org [Internet]. [cited 2026 Mar 6]. Life’s Essential 8. Available from: https://www.heart.org/en/healthy-living/healthy-lifestyle/lifes-essential-8

[6] Lloyd-Jones DM, Hong Y, Labarthe D, Mozaffarian D, Appel LJ, Van Horn L, et al. Defining and Setting National Goals for Cardiovascular Health Promotion and Disease Reduction: The American Heart Association’s Strategic Impact Goal Through 2020 and Beyond. Circulation. 2010;121(4):586–613. 10.1161/CIRCULATIONAHA.109.192703. 20089546 10.1161/CIRCULATIONAHA.109.192703

[7] Dhanasekara CS, Ancona D, Cortes L, Hu A, Rimu AH, Robohm-Leavitt C, et al. Association Between Autism Spectrum Disorders and Cardiometabolic Diseases: A Systematic Review and Meta-analysis. JAMA Pediatr. 2023;177(3):248–57. 10.1001/jamapediatrics.2022.5629. 36716018 10.1001/jamapediatrics.2022.5629 PMC9887535

[8] Chieh AY, Bryant BM, Kim JW, Li L. Systematic review investigating the relationship between autism spectrum disorder and metabolic dysfunction. Res Autism Spectr Disord. 2021;86:101821. 10.1016/j.rasd.2021.101821. 36570741 10.1016/j.rasd.2021.101821 PMC9784428

[9] Li Y, Xie T, Li L, Lin J, Vos M, Chang Z, et al. Cardiometabolic conditions in people with autism: a nationwide prospective cohort study from the Netherlands. Nat Ment Health. 2026;4(1):157–64. 10.1038/s44220-025-00546-9.

[10] Hand BN, Angell AM, Harris L, Carpenter LA. Prevalence of physical and mental health conditions in Medicare-enrolled, autistic older adults. Autism. 2020;24(3):755–64. 10.1177/1362361319890793. 31773968 10.1177/1362361319890793 PMC7433648

[11] Jin T, Huang W, Pang Q, Cao Z, Xing D, Guo S, et al. Genetically identified mediators associated with increased risk of stroke and cardiovascular disease in individuals with autism spectrum disorder. J Psychiatr Res. 2024;174:172–80. 10.1016/j.jpsychires.2024.04.027. 38640796 10.1016/j.jpsychires.2024.04.027

[12] Matheson BE, Douglas JM. Overweight and obesity in children with autism spectrum disorder (ASD): A critical review investigating the etiology, development, and maintenance of this relationship. Rev J Autism Dev Disord. 2017;4(2):142–56.

[13] Micai M, Fatta LM, Gila L, Caruso A, Salvitti T, Fulceri F, et al. Prevalence of co-occurring conditions in children and adults with autism spectrum disorder: A systematic review and meta-analysis. Neurosci Biobehav Rev. 2023;155:105436. 10.1016/j.neubiorev.2023.105436. 37913872 10.1016/j.neubiorev.2023.105436

[14] Rydzewska E, Dunn K, Cooper SA. Umbrella systematic review of systematic reviews and meta-analyses on comorbid physical conditions in people with autism spectrum disorder. Br J Psychiatry J Ment Sci. 2021;218(1):10–9. 10.1192/bjp.2020.167. 10.1192/bjp.2020.167 33161922

[15] Franklin BA, Eijsvogels TMH, Pandey A, Quindry J, Toth PP. Physical activity, cardiorespiratory fitness, and cardiovascular health: A clinical practice statement of the ASPC Part I: Bioenergetics, contemporary physical activity recommendations, benefits, risks, extreme exercise regimens, potential maladaptations. Am J Prev Cardiol. 2022;12:100424. 10.1016/j.ajpc.2022.100424. 36281324 10.1016/j.ajpc.2022.100424 PMC9586848

[16] Hillier A, Buckingham A, SchenaII D. Physical Activity Among Adults With Autism: Participation, Attitudes, and Barriers. Percept Mot Skills. 2020;127(5):874–90. 10.1177/0031512520927560. 32443953 10.1177/0031512520927560

[17] Jachyra P, Renwick R, Gladstone B, Anagnostou E, Gibson BE. Physical activity participation among adolescents with autism spectrum disorder. Autism. 2021;25(3):613–26. 10.1177/1362361320949344. 32921151 10.1177/1362361320949344

[18] Blagrave AJ, Colombo-Dougovito AM, Healy S. Just Invite Us: Autistic Adults’ Recommendations for Developing More Accessible Physical Activity Opportunities. Autism Adulthood. 2021;3(2):179–86. 10.1089/aut.2020.0055. 36601469 10.1089/aut.2020.0055 PMC8992896

[19] Lee D, Kennedy J, Cothran DJ, Shih PC, Dickinson S, Golzarri-Arroyo L, et al. Correlates of physical activity, sedentary time, and cardiovascular disease risk factors in autistic adults without intellectual disabilities. Res Dev Disabil. 2025;161:104980. 10.1016/j.ridd.2025.104980. 40138869 10.1016/j.ridd.2025.104980

[20] Charlier L, Cordeiro L, Neto JLC, Signini ÉDF, Barbosa-Silva J, Corbellini C, et al. Effects of physical exercise on cardiometabolic health in individuals with autism spectrum disorder: A systematic review. Healthcare. 2025;13(4). 10.3390/healthcare13040439. 10.3390/healthcare13040439 PMC11855590 39997314

[21] Ballester P, Martínez MJ, Javaloyes A, Inda MdelM, Fernández N, Gázquez, et al. Sleep problems in adults with autism spectrum disorder and intellectual disability. Autism Res. 2019;12(1):66–79. 10.1002/aur.2000. 30273974 10.1002/aur.2000

[22] Jackson CL, Redline S, Emmons KM. Sleep as a potential fundamental contributor to disparities in cardiovascular health. Annu Rev Public Health. 2015;36:417–40. . 10.1146/annurev-publhealth-031914-122838 25785893 10.1146/annurev-publhealth-031914-122838 PMC4736723

[23] Bishop L, Charlton RA, McLean KJ, McQuaid GA, Lee NR, Wallace GL. Cardiovascular disease risk factors in autistic adults: The impact of sleep quality and antipsychotic medication use. Autism Res. 2023;16(3):569–79. 10.1002/aur.2872. 36490360 10.1002/aur.2872 PMC10023317

[24] Kaar JL, Luberto CM, Campbell KA, Huffman JC. Sleep, health behaviors, and behavioral interventions: Reducing the risk of cardiovascular disease in adults. World J Cardiol. 2017;9(5):396–406. 10.4330/wjc.v9.i5.396. 28603586 10.4330/wjc.v9.i5.396 PMC5442407

[25] Fekete M, Szarvas Z, Fazekas-Pongor V, Feher A, Csipo T, Forrai J, et al. Nutrition strategies promoting healthy aging: From improvement of cardiovascular and brain health to prevention of age-associated diseases. Nutrients. 2022;15(1). 10.3390/nu15010047. 10.3390/nu15010047 PMC9824801 36615705

[26] Remón S, Ferrer-Mairal A, Sanclemente T, Remón S, Ferrer-Mairal A, Sanclemente T. Food and nutrition in autistic adults: Knowledge gaps and future perspectives. Nutrients. 2025;17(9). 10.3390/nu17091456. 10.3390/nu17091456 PMC12073154 40362765

[27] Adams JB, Audhya T, Geis E, Gehn E, Fimbres V, Pollard EL, et al. Comprehensive nutritional and dietary intervention for autism spectrum disorder—a randomized, controlled 12-month trial. Nutrients. 2018;10(3). 10.3390/nu10030369. 10.3390/nu10030369 PMC5872787 29562612

[28] Al-Beltagi M. Nutritional management and autism spectrum disorder: A systematic review. World J Clin Pediatr. 2024;13(4):99649. 10.5409/wjcp.v13. 39654662 10.5409/wjcp.v13.i4.99649 PMC11572612

[29] Buro AW, Gray HL, Kirby RS, Marshall J, Strange M, Hasan S, et al. Pilot Study of a Virtual Nutrition Intervention for Adolescents and Young Adults With Autism Spectrum Disorder. J Nutr Educ Behav. 2022;54(9):853–62. 10.1016/j.jneb.2022.01.008. 36087955 10.1016/j.jneb.2022.01.008 PMC10164280

[30] Sathe N, Andrews JC, McPheeters ML, Warren ZE. Nutritional and Dietary Interventions for Autism Spectrum Disorder: A Systematic Review. Pediatrics. 2017;139(6):e20170346. 10.1542/peds.2017-0346. 28562286 10.1542/peds.2017-0346

[31] Tomiyama AJ. Stress and obesity. Annu Rev Psychol. 2019;70:703–18. 29927688 10.1146/annurev-psych-010418-102936

[32] Razzoli M, Bartolomucci A. The Dichotomous Effect of Chronic Stress on Obesity. Trends Endocrinol Metab. 2016;27(7):504–15. 10.1016/j.tem.2016.04.007. 27162125 10.1016/j.tem.2016.04.007 PMC4912918

[33] Siddiqui NZ, Beulens JWJ, van der Vliet N, den Braver NR, Elders PJM, Rutters F. The longitudinal association between chronic stress and (visceral) obesity over seven years in the general population: The Hoorn Studies. Int J Obes. 2022;1–10. 10.1038/s41366-022-01179-z. 10.1038/s41366-022-01179-z 35851315

[34] Gianotti L, Belcastro S, D’Agnano S, Tassone F. The Stress Axis in Obesity and Diabetes Mellitus: An Update. Endocrines. 2021;2(3):3. 10.3390/endocrines2030031.

[35] Cotter EW, Kelly NR. Stress-related eating, mindfulness, and obesity. Health Psychol. 2018;37(6):516–25. 10.1037/hea0000614. 29708389 10.1037/hea0000614 PMC6488023

[36] Wiss DA, Brewerton TD. Adverse Childhood Experiences and Adult Obesity: A Systematic Review of Plausible Mechanisms and Meta-Analysis of Cross-Sectional Studies. Physiol Behav. 2020;223:112964. 10.1016/j.physbeh.2020.112964. 32479804 10.1016/j.physbeh.2020.112964

[37] Valderhaug TG, Slavich GM. Assessing Life Stress: A Critical Priority in Obesity Research and Treatment. Obesity. 2020;28(9):1571–3. 10.1002/oby.22911. 32729167 10.1002/oby.22911 PMC7483345

[38] Ruiz LD, Zuelch ML, Dimitratos SM, Scherr RE. Adolescent Obesity: Diet Quality, Psychosocial Health, and Cardiometabolic Risk Factors. Nutrients. 2020;12(1):1. 10.3390/nu12010043. 10.3390/nu12010043 PMC7020092 31877943

[39] Engel GL. The Need for a New Medical Model: A Challenge for Biomedicine. Science. 1977;196(4286):129–36. 10.1126/science.847460. 847460 10.1126/science.847460

[40] Aubé B, Follenfant A, Goudeau S, Derguy C. Public Stigma of Autism Spectrum Disorder at School: Implicit Attitudes Matter. J Autism Dev Disord. 2021;51(5):1584–97. 10.1007/s10803-020-04635-9. 32780195 10.1007/s10803-020-04635-9

[41] Botha M, Dibb B, Frost DM. Autism is me: an investigation of how autistic individuals make sense of autism and stigma. Disabil Soc. 2020;0(0):1–27. 10.1080/09687599.2020.1822782.

[42] Clark LH, Hudson JL, Haider T. Anxiety specific mental health stigma and help-seeking in adolescent males. J Child Fam Stud. 2020;1–12.

[43] Grinker RR. Autism,stigma, disability: a shifting historical terrain. Curr Anthropol. 2020;61(S21):S55–67.

[44] Gillespie-Lynch K, Daou N, Obeid R, Reardon S, Khan S, Goldknopf EJ. What Contributes to Stigma Towards Autistic University Students and Students with Other Diagnoses? J Autism Dev Disord. 2021;51(2):459–75. 10.1007/s10803-020-04556-7. 32504342 10.1007/s10803-020-04556-7 PMC7273383

[45] Pecora LA, Hancock GI, Mesibov GB, Stokes MA. Characterising the Sexuality and Sexual Experiences of Autistic Females. J Autism Dev Disord. 2019;49(12):4834–46. 10.1007/s10803-019-04204-9. 31463632 10.1007/s10803-019-04204-9

[46] Reuben KE, Stanzione CM, Singleton JL. Interpersonal trauma and posttraumatic stress in autistic adults. Autism Adulthood. 2021. 10.1089/aut.2020.0073 PMC8992908 36605371

[47] Moseley RL, Turner-Cobb JM, Spahr CM, Shields GS, Slavich GM. Lifetime and perceived stress, social support, loneliness, and health in autistic adults. Health Psychol. 2021;40:556–68. 10.1037/hea0001108. 34618502 10.1037/hea0001108 PMC8513810

[48] Kim SA, Baczewski L, Pizzano M, Kasari C, Sturm A. Discrimination and harassment experiences of autistic college students and their neurotypical peers: Risk and protective factors. J Autism Dev Disord. 2022;14. 10.1007/s10803-022-05729-2. 10.1007/s10803-022-05729-2 PMC10627989 36103077

[49] van der Lubbe A, Swaab H, van den Akker E, Vermeiren R, Ester WA. Hair cortisol in young children with autism and their parents: Associations with child mental health, eating behavior and weight status. J Autism Dev Disord. 2025;22. 10.1007/s10803-024-06672-0. 10.1007/s10803-024-06672-0 PMC13222298 39841400

[50] van der Lubbe A, Swaab H, Vermeiren R, van Rossum EFC, van Balkom IDC, Ester WA. Chronic parenting stress in parents of children with autism: Associations with chronic stress in their child and parental mental and physical health. J Autism Dev Disord. 2025;21. 10.1007/s10803-025-06736-9. 10.1007/s10803-025-06736-9 PMC13346221 39982675

[51] Chen X, Gelaye B, Velez JC, Barbosa C, Pepper M, Andrade A, et al. Caregivers’ hair cortisol: a possible biomarker of chronic stress is associated with obesity measures among children with disabilities. BMC Pediatr. 2015;15(1):9. 10.1186/s12887-015-0322-y. 25886364 10.1186/s12887-015-0322-y PMC4339433

[52] Dijkstra-de Neijs L, Leenen PJM, Hays JP, van der Valk ES, Kraaij R, van Rossum EFC, et al. Biological Consequences of Psychological Distress in Caregivers of Children with Autism Spectrum Disorder and its Potential Relevance to Other Chronic Diseases Including Cancer. Curr Epidemiol Rep. 2020;7(3):139–48. 10.1007/s40471-020-00237-2.

[53] Hotez E, Tsevat RK, Tao S, Phan JM, Smith P, Shen T, et al. Autism, obesity, and ptsd among adolescents and young adults: An analysis of national medicaid claims data. J Autism Dev Disord. 2025;17. 10.1007/s10803-025-06881-1. 10.1007/s10803-025-06881-1 PMC12434421 40381092

[54] Edmondson D, von Känel R. Post-traumatic stress disorder and cardiovascular disease. Lancet Psychiatry. 2017;4(4):320–9. 10.1016/S2215-0366(16)30377-7. 28109646 10.1016/S2215-0366(16)30377-7 PMC5499153

[55] O’Donnell CJ, Schwartz Longacre L, Cohen BE, Fayad ZA, Gillespie CF, Liberzon I, et al. Posttraumatic Stress Disorder and Cardiovascular Disease: State of the Science, Knowledge Gaps, and Research Opportunities. JAMA Cardiol. 2021;6(10):1207–16. 10.1001/jamacardio.2021.2530. 34259831 10.1001/jamacardio.2021.2530

[56] Jain A, Davis AM. Primary Prevention of Cardiovascular Disease. JAMA. 2019;322(18):1817–8. 10.1001/jama.2019.15915. 31584604 10.1001/jama.2019.15915

[57] Gilmore D, Krantz M, Weaver L, Hand BN. Healthcare service use patterns among autistic adults: A systematic review with narrative synthesis. Autism. 2022;26(2):317–31. 10.1177/13623613211060906. 34881676 10.1177/13623613211060906

[58] Mason D, Ingham B, Urbanowicz A, Michael C, Birtles H, Woodbury-Smith M, et al. A Systematic Review of What Barriers and Facilitators Prevent and Enable Physical Healthcare Services Access for Autistic Adults. J Autism Dev Disord. 2019;49(8):3387–400. 10.1007/s10803-019-04049-2. 31124030 10.1007/s10803-019-04049-2 PMC6647496

[59] Hotez E, Duan S, Ma J, Shankar S. An Urgent Need to Improve Accessibility of Preventive Services for People With Disabilities. Ann Intern Med. 2025;178(5):741–2. 10.7326/ANNALS-25-00374. 40063958 10.7326/ANNALS-25-00374

[60] McCormack G, Dillon AC, Healy O, Walsh C, Lydon S. Primary Care Physicians’ Knowledge of Autism and Evidence-Based Interventions for Autism: A Systematic Review. Rev J Autism Dev Disord. 2020;7(3):226–41. 10.1007/s40489-019-00189-4.

[61] Ames JL, Massolo ML, Davignon MN, Qian Y, Cerros HJ, Croen LA. Transitioning youth with autism spectrum disorders and other special health care needs into adult primary care: A provider survey. Autism. 2021;25(3):731–43. 10.1177/1362361320926318. 32551940 10.1177/1362361320926318

[62] Nicolaidis C, Raymaker DM, Ashkenazy E, McDonald KE, Dern S, Baggs AE, et al. Respect the way I need to communicate with you: Healthcare experiences of adults on the autism spectrum. Autism. 2015;19(7):824–31. 10.1177/1362361315576221. 25882392 10.1177/1362361315576221 PMC4841263

[63] Best M, Lindsay R, Demissie S, Burakov I, Ramos-Torres S, Burke M. From awareness to action: Facilitators and advocacy in healthcare by autistic adults. Autism. 2025;29(10):2513–23. 10.1177/13623613251343350. 40421583 10.1177/13623613251343350 PMC12417605

[64] Chen MH, Lan WH, Hsu JW, Huang KL, Su TP, Li CT, et al. Risk of Developing Type 2 Diabetes in Adolescents and Young Adults With Autism Spectrum Disorder: A Nationwide Longitudinal Study. Diabetes Care. 2016;39(5):788–93. 10.2337/dc15-1807. 27006513 10.2337/dc15-1807

[65] Byrne K, Sterrett K, Elias R, Bal VH, McCauley JB, Lord C. Trajectories of Seizures, Medication Use, and Obesity Status into Early Adulthood in Autistic Individuals and Those with Other Neurodevelopmental Conditions. Autism Adulthood. 2022;4(2):110–9. 10.1089/aut.2020.0080. 36605975 10.1089/aut.2020.0080 PMC9242707

[66] Croteau C, Ben Amor L, Ilies D, Mottron L, Tarride JE, Dorais M, et al. Impact of Psychoactive Drug Use on Developing Obesity among Children and Adolescents with Autism Spectrum Diagnosis: A Nested Case–Control Study. Child Obes. 2019;15(2):131–41. 10.1089/chi.2018.0170. 30668140 10.1089/chi.2018.0170

[67] Healy S, Aigner CJ, Haegele JA. Prevalence of overweight and obesity among US youth with autism spectrum disorder. Autism. 2019;23(4):1046–50. 10.1177/1362361318791817. 30101597 10.1177/1362361318791817

[68] Schott W, Tao S, Shea L. Co-occurring conditions and racial-ethnic disparities: Medicaid enrolled adults on the autism spectrum. Autism Res. 2022;15(1):70–85. 10.1002/aur.2644. 34854249 10.1002/aur.2644 PMC8812993

[69] DaWalt LS, Taylor JL, Movaghar A, Hong J, Kim B, Brilliant M, et al. Health profiles of adults with autism spectrum disorder: Differences between women and men. Autism Res. 2021;14(9):1896–904. 10.1002/aur.2563. 34213066 10.1002/aur.2563 PMC8592037

[70] Weir E, Allison C, Warrier V, Baron-Cohen S. Increased prevalence of non-communicable physical health conditions among autistic adults. Autism. 2021;25(3):681–94. 10.1177/1362361320953652. 32907337 10.1177/1362361320953652 PMC7610707

[71] Bishop L, Charlton RA, McLean KJ, McQuaid GA, Lee NR, Wallace GL. Cardiovascular Disease Risk Factors in Autistic Adults: The Impact of Sleep Quality and Antipsychotic Medication Use. Autism Res Off J Int Soc Autism Res. 2023;16(3):569–79. 10.1002/aur.2872. 10.1002/aur.2872 PMC10023317 36490360

[72] Malow BA, Qian Y, Ames JL, Alexeeff S, Croen LA. Health conditions in autism: Defining the trajectory from adolescence to early adulthood. Autism Res. 2023;16(7):1437–49. 10.1002/aur.2960. 37377040 10.1002/aur.2960 PMC10524876

[73] Byrne K, Sterrett K, Elias R, Bal VH, McCauley JB, Lord C. Trajectories of seizures, medication use, and obesity status into early adulthood in autistic individuals and those with other neurodevelopmental conditions. Autism Adulthood. 2021. 10.1089/aut.2020.0080 10.1089/aut.2020.0080 PMC9242707 36605975

[74] Buro AW, Salinas-Miranda A, Marshall J, Gray HL, Kirby RS. Correlates of obesity in adolescents with and without autism spectrum disorder: The 2017–2018 National Survey of Children’s Health. Disabil Health J. 2022;15(2):101221. 10.1016/j.dhjo.2021.101221. 34654677 10.1016/j.dhjo.2021.101221

[75] Fuhrmann D, Knoll LJ, Blakemore SJ. Adolescence as a Sensitive Period of Brain Development. Trends Cogn Sci. 2015;19(10):558–66. 10.1016/j.tics.2015.07.008. 26419496 10.1016/j.tics.2015.07.008

[76] Dahl RE, Allen NB, Wilbrecht L, Suleiman AB. Importance of investing in adolescence from a developmental science perspective. Nature. 2018;554(7693):7693. 10.1038/nature25770. 10.1038/nature25770 29469094

[77] Yacamán-Méndez D, Trolle-Lagerros Y, Zhou M, Monteiro Ponce de Leon A, Gudjonsdottir H, Tynelius P, et al. Life-course trajectories of weight and their impact on the incidence of type 2 diabetes. Sci Rep. 2021;11(1):12494. 10.1038/s41598-021-91910-z. 34127722 10.1038/s41598-021-91910-z PMC8203741

[78] Fortuna RJ, Robinson L, Smith TH, Meccarello J, Bullen B, Nobis K, et al. Health Conditions and Functional Status in Adults with Autism: A Cross-Sectional Evaluation. J Gen Intern Med. 2016;31(1):77–84. 10.1007/s11606-015-3509-x. 26361965 10.1007/s11606-015-3509-x PMC4700008

[79] Must A, Eliasziw M, Phillips SM, Curtin C, Kral TVE, Segal M, et al. The Effect of Age on the Prevalence of Obesity among US Youth with Autism Spectrum Disorder. Child Obes. 2017;13(1):25–35. 10.1089/chi.2016.0079. 27704874 10.1089/chi.2016.0079 PMC5278796

[80] Healy S, Pacanowski CR, Williams E. Weight management interventions for youth with autism spectrum disorder: a systematic review. Int J Obes. 2019;43(1):1. 10.1038/s41366-018-0233-8. 10.1038/s41366-018-0233-8 30305689

[81] Broder-Fingert S, Brazauskas K, Lindgren K, Iannuzzi D, Van Cleave J. Prevalence of Overweight and Obesity in a Large Clinical Sample of Children With Autism. Acad Pediatr. 2014;14(4):408–14. 10.1016/j.acap.2014.04.004. 24976353 10.1016/j.acap.2014.04.004

[82] Buro AW, Salinas-Miranda A, Marshall J, Gray HL, Kirby RS. Obesity and co-occurring conditions among adolescents with autism spectrum disorder: The National Survey of Children’s Health 2017–2018. Res Autism Spectr Disord. 2022;92:101927. 10.1016/j.rasd.2022.101927.

[83] Hill AP, Zuckerman KE, Fombonne E. Obesity and Autism. Pediatrics. 2015;136(6):1051–61. 10.1542/peds.2015-1437. 26527551 10.1542/peds.2015-1437 PMC4657601

[84] Kahathuduwa CN, West BD, Blume J, Dharavath N, Moustaid-Moussa N, Mastergeorge A. The risk of overweight and obesity in children with autism spectrum disorders: A systematic review and meta-analysis. Obes Rev. 2019;20(12):1667–79. 31595678 10.1111/obr.12933

[85] PhillipsSarah M, KralTanja VE, SherwoodNancy E, StanishHeidi I, BandiniLinda G. The effect of age on the prevalence of obesity among US youth with autism spectrum disorder. Child Obes. 2017. 10.1089/chi.2016.0079 PMC5278796 27704874

[86] Voulgarakis H, Bendell-Estroff D, Field T. Prevalence of obesity and autism spectrum disorder. Behav Dev Bull. 2017;22(1):209. 10.1037/bdb0000057 PMC5476317 28642808

[87] Zheng Z, Zhang L, Li S, Zhao F, Wang Y, Huang L, et al. Association among obesity, overweight and autism spectrum disorder: a systematic review and meta-analysis. Sci Rep. 2017;7(1):1. 10.1038/s41598-017-12003-4. 28916794 10.1038/s41598-017-12003-4 PMC5601947

[88] Mohammedsaeed W, El Shikieri AB, Alharbi MF. Exploring the link between exercise, BMI, cardiometabolic health, glycemic control, and health outcomes in autistic children: A comprehensive investigation. Res Autism. 2025;127:202665. 10.1016/j.reia.2025.202665.

[89] Jin C, Shi W. Considerations Regarding Autism Spectrum Disorders and Cardiometabolic Diseases. JAMA Pediatr. 2023;177(7):737. 10.1001/jamapediatrics.2023.1022. 37155162 10.1001/jamapediatrics.2023.1022

[90] Frye RE, Vassall S, Kaur G, Lewis C, Karim M, Rossignol D. Emerging biomarkers in autism spectrum disorder: a systematic review. Ann Transl Med. 2019;7(23):792. 10.21037/atm.2019.11.53. 32042808 10.21037/atm.2019.11.53 PMC6989979

[91] Buckley DI, Hsu F, Dana T, Blackie K, Holmes R, Nygren P, et al. Health Care Delivery of Clinical Preventive Services for People With Disabilities. Ann Intern Med. 2025;178(5):671–86. 10.7326/ANNALS-24-02446. 40063961 10.7326/ANNALS-24-02446

[92] Weir EM, Autism. Physical Health Conditions, and a Need for Reform. JAMA Pediatr. 2023;177(3):229–30. 10.1001/jamapediatrics.2022.5639. 36716011 10.1001/jamapediatrics.2022.5639

[93] Morris R, Greenblatt A, Saini M. Healthcare Providers’ Experiences with Autism: A Scoping Review. J Autism Dev Disord. 2019;49(6):2374–88. 10.1007/s10803-019-03912-6. 30758692 10.1007/s10803-019-03912-6

[94] Ames JL, Massolo ML, Davignon MN, Qian Y, Croen LA. Healthcare service utilization and cost among transition-age youth with autism spectrum disorder and other special healthcare needs. Autism. 2021;25(3):705–18. 10.1177/1362361320931268. 32583679 10.1177/1362361320931268

[95] Mazurek MO, Stobbe G, Loftin R, Malow BA, Agrawal MM, Tapia M, et al. ECHO Autism Transition: Enhancing healthcare for adolescents and young adults with autism spectrum disorder. Autism. 2020;24(3):633–44. 10.1177/1362361319879616. 31581793 10.1177/1362361319879616

[96] Haas K, Costley D, Falkmer M, Richdale A, Sofronoff K, Falkmer T. Factors Influencing the Research Participation of Adults with Autism Spectrum Disorders. J Autism Dev Disord. 2016;46(5):1793–805. 10.1007/s10803-016-2708-6. 26810436 10.1007/s10803-016-2708-6

[97] Le Cunff AL, Glover C, Martis BL, Giampietro V, Dommett E. Methodological adjustments for experimental studies including neurodiverse participants: A checklist for before, during, and after laboratory visits. MethodsX. 2024;12:102658. 10.1016/j.mex.2024.102658. 38510933 10.1016/j.mex.2024.102658 PMC10950872

[98] Rios D, Magasi S, Novak C, Harniss M. Conducting Accessible Research: Including People With Disabilities in Public Health, Epidemiological, and Outcomes Studies. Am J Public Health. 2016;106(12):2137–44. 10.2105/AJPH.2016.303448. 27736212 10.2105/AJPH.2016.303448 PMC5104996

